# A Three-Dimensional Shape-Based Force and Stiffness-Sensing Platform for Tendon-Driven Catheters

**DOI:** 10.3390/s16070990

**Published:** 2016-06-28

**Authors:** Minou Kouh Soltani, Sohrab Khanmohammadi, Farzan Ghalichi

**Affiliations:** 1 Department of Electrical and Computer Engineering, University of Tabriz, Tabriz 51666-14766, Iran; kouhsoltni@tabrizu.ac.ir; 2 Department of Biomedical Engineering, Sahand University of Technology, Tabriz 51335-1996, Iran; fghalichi@sut.ac.ir

**Keywords:** catheters, force sensing, stiffness estimation, iterative, nonlinear Kalman filter, adaptive

## Abstract

This paper presents an efficient shape-based three-axial force and stiffness estimator for active catheters commonly implemented in cardiac ablation. The force-sensing capability provides important feedback for catheterization procedures including real-time control and catheter steering in autonomous navigation systems. The proposed platform is based on the introduced accurate and computationally efficient Cosserat rod model for tendon-driven catheters. The proposed nonlinear Kalman filter formulation for contact force estimation along with the developed catheter model provides a real-time force observer robust to nonlinearities and noise covariance uncertainties. Furthermore, the proposed platform enables stiffness estimation in addition to tip contact force sensing in different operational circumstances. The approach incorporates pose measurements which can be achieved using currently developed pose-sensing systems or imaging techniques. The method makes the approach compatible with the range of forces applied in clinical applications. The simulation and experimental results verify the viability of the introduced force and stiffness-sensing technique.

## 1. Introduction

**Figure 1:**
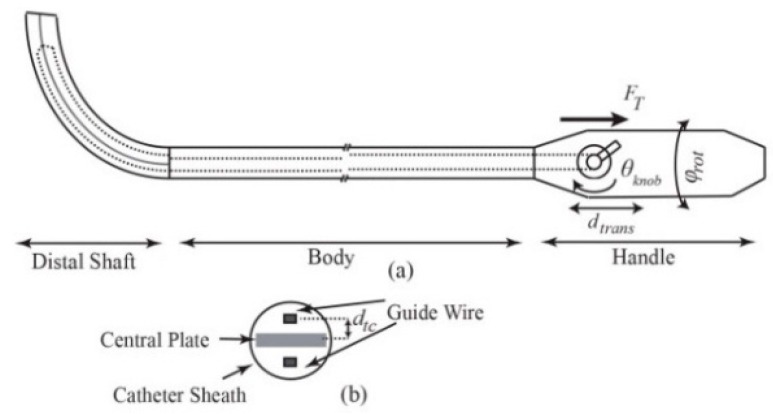
The schematic representation of the ablation catheter: (**a**) Tendons are represented by dashed lines and the central plate (thin plate tendons attached to it) is depicted with solid lines inside the distal shaft. $\theta_{knob},\;\varphi_{rot}$ and $d_{trans}$ are representing steering knob rotation, handle twist and translation, respectively; (**b**) Cross-section of the distal shaft.

Many medical interventions such as cardiac interventions and interventional radiology involve deployment of steerable catheters for diagnosis and treatment of internal body organs [1,2]. Despite recent advances in robot-assisted catheterization [2,3,4,5,6], the catheterization is usually performed manually by rotating and translating the proximal end of the catheter as well as bending the distal shaft via a steering knob on the handle (Figure 1). Steering of catheters, due to their inherent flexibility and operation in a confined environment, requires high dexterity and expertise to navigate and place the tip on the target tissue. Tip force measurement could have great implications in the success of both manual and robot-assisted procedures. For instance, during the cardiac ablation, a compliance force should be applied by the end point to the intra-cardiac tissue for a certain period of time and the tip of catheter must maintain a stable contact with the target, whereas inadequate force monitoring results in complications, including perforation and permanent destruction of cardiac tissues due to excessive force application or recurrence of the cardiac problem owing to low force implementation [1,7,8].

Despite its importance, force feedback remains one of the foremost challenges in advancing catheterization techniques. To this end, two commercial tri-axial force-sensing ablation catheters have been recently introduced, namely TactiCath^®^ catheter system (St. Jude Medical, St. Paul, MN, USA) and Thermocool^®^ SmartTouch™ (Biosense Webster, Diamond Bar, CA, USA) [9,10]. The TactiCath^®^ is a 3.5-mm open-irrigated tip radiofrequency (RF) ablation catheter which uses tri-axial fiberoptic cable for contact force sensing. The Thermocool^®^ SmartTouch™ has a 3.5-mm six-hole open-irrigated tip that measures the force by deformation of a precision spring which is located between the tip and distal shaft of the catheter. Both catheters measure and visualize the magnitude and direction of tip contact forces, but they require accompanying systems to operate with. Therefore, equipping the operating room with the specialized navigation system in addition to the higher cost of the force-sensing catheters impose significant limitations to their use. Some recent studies have focused on the development of sensors and catheters with force sensors, including Polygerinos et al., who developed three-dimensional magnetic resonance imaging (MRI)-compatible fiber optic force sensor with the reported force resolution of 0.01 N and 5% error in force measurement with the maximum magnitude of 0.5 N [11]. Kenser and Howe developed an axial fiber optic force sensor and mounted it on the ablation catheter tip [12,13]. The prototype is approximately 15Fr (5 mm) in diameter with 2%–4% error in axial force sensing. 

A multi-axis force sensor at the tip of the tool directly measures the interaction force. However, the introduced sensors suffer from significant impediments. They are limited in the number of measurement axes, and introduce extra cost, packaging issues, sensitivity to temperature, and limitations in MRI compatibility. Integration of the sensor with conventional systems may also lead to interference with the operation of the ablation catheters, and create an extra obstacle to the effort for producing smaller end-effectors. To cope with these problems, the tip-tissue contact force would ideally be estimated without using force sensors.

Xu et al. presented the idea of “intrinsic force sensing” for continuum robots that is based on the evaluation of certain components of tip forces through the joint actuation loads [14], and introduced performance index for analysis of full-wrench sensing [15]. Inspired by the suggested concept, Rucker and Webster proposed a probabilistic approach for planar continuum robot force estimation using tip pose and actuation torques [16]. However, their method is limited to planar forces and suffers from convergence issues. Khoshnam et al. investigated contact force estimation with the Euler-Bernouli model for the catheter deflection [17], where the planar contact forces were estimated using a strain sensor mounted at the distal base and numerical mapping of the tip angles to the sensor readings. In a recent study [18], it was demonstrated that the flexibility of a catheter’s distal shaft can be implemented as a means of tip contact force estimation. To this end, the same group introduced the force index and provided only the contact force range with 80% accuracy. In summary, the aforementioned approaches have one or more of the following limitations: assumption of planar forces/deformation, small deformations, and constant curvature or piece-wise constant curvature, in addition to convergence issues, sensitivity to noise, and need for actuation torque measurements.

To address the aforementioned problems, this paper contributes by developing a 3D force estimation platform for tendon-driven catheters. For this purpose, we introduced the more powerful Cosserat rod-based quasi-static model of tendon-driven catheters that can be used for both small and large 3D deformations. The computational efficiency of the model along with its accuracy makes it appealing for real-time applications. Based on the model, tri-axial shape-based contact force and stiffness estimators for tendon-driven catheters are developed which do not require actuation loading information. Potential for stiffness estimation is another advantage of the model. Tip pose and shape measurements, readily available in clinical applications through imaging, can be deployed for tip contact force estimation avoiding extra sensor integration [19,20]. In this study, electromagnetic tracking sensors were incorporated for pose measurements. Several force estimation platforms based on the Kalman filter (KF) are designed and their performances are compared in terms of efficiency, convergence, and robustness. The adoption of a KF-based approach makes it less sensitive to noise and enables next time step force prediction (useful for tracking control purposes).

## 2. Modeling the Steerable Catheter 

A model of force displacement is required for shape-based force estimation of tendon-driven catheters. Therefore, with the introduction of the Cosserat theory of elastic rods [21,22,23], we develop the quasi-static model for tendon-driven intra-cardiac catheters. For convenience, the nomenclature is introduced in Table 1.

### 2.1. Kinematics

**Figure 2:**
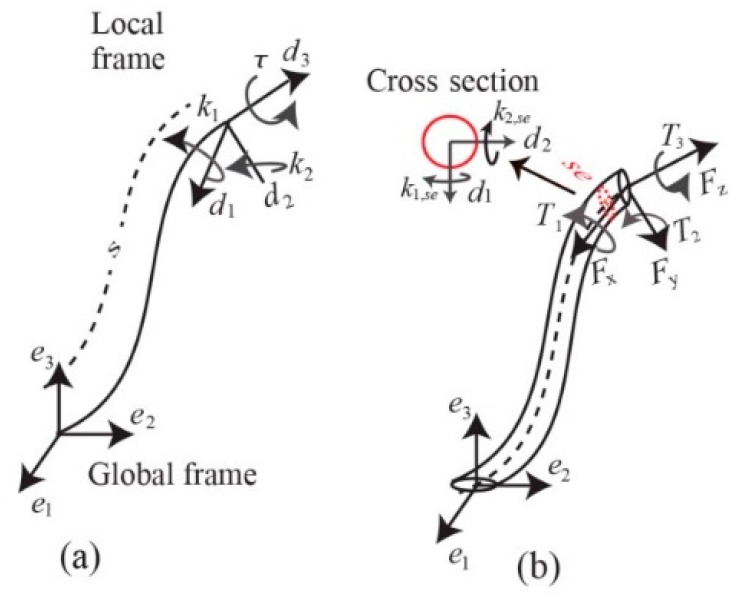
(**a**) Kinematics of the nonlinear rod; (**b**) Catheter modeled as an elastic rod.

An elastic rod with an unstretched length *L* is characterized by the global coordinate frame $e=\left\{e_{1},e_{2},e_{3}\right\}$ represented in Figure 2a. The vector **r**(*s*), s∈[0,L] locates the centerline of the rod with respect to the global coordinate. The local frame $d(s)=\left\{d_{1}(s),d_{2}(s),d_{3}(s)\right\}$ attached to the curve along the centerline demonstrates the orientation $R_{c}(s)\in SO(3)$ of each point on the rod relative to the global frame. The director $d_{3}$ is adopted to be tangent to the centerline at each infinitesimal segment of the rod and $d_{1}$ and $d_{2}$ define the local frame on the cross-section of the rod. The velocity vector $v^{l}={\left[v_{1}^{l},v_{2}^{l},v_{3}^{l}\right]}^{T}$ defines shear strain along $d_{1}$ and $d_{2}$ by the first two components, and axial compression or stretch along $d_{3}$ by $v_{3}^{l}$, where superscript *l* indicates definition with respect to the local frame. With the shear strain and elongation being negligible, the change of the rod’s position with respect to arc length becomes,

(1) $${\dot{\text{r}}}^{l}(s)=d_{3}$$

The rate of change of each director along arc length is,

(2) $${\dot{\text{d}}}_{i}=\omega \times d_{i},\;i=1,2,3$$

where the Darboux vector is given by $\omega ={\left[k_{1},k_{2},\tau \right]}^{T}$ with $k_{i}\;(i=1,2),$ denoting curvature along $d_{i}$ and τ representing the torsion.

### 2.2. Governing Equilibrium Equations

The governing equilibrium equation of model in global frame is described by

(3) $$\begin{array}{l} \dot{\text{n}}(s)+f(s)=0\\ \dot{\text{m}}(s)+\dot{\text{r}}(s)\times n(s)+l(s)=0 \end{array}$$

where dot represents the derivative with respect to arc length [22].

The internal moment and force vectors, represented by $m^{l}(s)={\left[m_{1}^{l}\;m_{2}^{l}\;m_{3}^{l}\right]}^{T}$ and $n^{l}(s)={\left[n_{1}^{l}\;n_{2}^{l}\;n_{3}^{l}\right]}^{T},$ respectively, are described with respect to the local frame. Internal moments are related to the local kinematic displacement of the rod as follows,

(4) $$m_{1}^{l}(s)=EI_{1}(k_{1}(s)-k_{1,0}),\;m_{2}^{l}(s)=EI_{2}(k_{2}(s)-k_{2,0}),\;m_{3}^{l}(s)=GJ_{s}(\tau (s)-\tau_{0})$$

### 2.3. Applied External Loads to the Ablation Catheter

The primary interacting forces for a tendon-driven catheter include tendon tension and cardiac-interacting load. 

The tendon tension force, $F_{T},$ leads to external torque $T^{l}=F_{T}d_{tc}$ at the tip along $d_{1}$ resulting from the distance $d_{tc}$ between the tendon and centerline (Figure 1b). This initial torque can be modeled as an initial curvature, $k_{1,0}$, as well [24,25].

During the ablation, consistent contact with the cardiac wall is required. The contact changes as a result of heartbeat and respiration that leads to deviation in the contact forces. Assuming that the contact force is not available, the shape and deflection of the catheter should be measured continuously to give the estimate of changing forces.

The underlying assumptions are as follows,

1. Gravity effect due to the low mass and inertia of distal shaft is ignored.
2. Blood drag force is negligible when compared to the tip contact and tension forces [23,26].
3. Tendon actuation effect is modeled as an end-moment load implemented at the tip of the distal shaft. In this regard, it is assumed that tendon’s axial tip force and distributed forces cancel each other, and the range of transverse tip forces and curvatures do not lead to significant model inaccuracy [23,27,28].

Therefore, we assume f(s)=l(s)=0, and consider the external point forces F to be applied at the tip of the rod.

### 2.4. Quasi-Static Model of Tendon-Driven Catheter

By substituting Equation (2) into Equation (3), the resulting internal force equilibrium equation is obtained. For ease of notation, dependence on *s* is dropped hereafter. Therefore, we have

(5) $$\begin{array}{l} \dot{n_{1}^{l}}+k_{2}n_{3}^{l}-\tau n_{2}^{l}=0\\ \dot{n_{2}^{l}}+\tau n_{1}^{l}-k_{1}n_{3}^{l}=0\\ \dot{n_{3}^{l}}+k_{1}n_{2}^{l}-k_{2}n_{1}^{l}=0 \end{array}$$

Likewise, replacing Equation (2) into Equation (3), and deploying Equations (1) and (4) leads to:

(6) $$\begin{array}{l} EI_{1}\dot{k_{1}}+(GJ_{s}-EI_{2})k_{2}\tau +\tau EI_{2}k_{2,0}-k_{2}GJ_{s}\tau_{0}-n_{2}^{l}=0\\ EI_{2}\dot{k_{2}}+(EI_{1}-GJ_{s})k_{1}\tau +k_{1}GJ_{s}\tau_{0}-\tau EI_{1}k_{1,0}+n_{1}^{l}=0\\ GJ_{s}\dot{\tau }+(EI_{2}-EI_{1})k_{1}k_{2}+k_{2}EI_{1}k_{1,0}-k_{1}EI_{2}k_{2,0}=0 \end{array}$$

The contact forces $n^{l}(l)={\left[F_{x}\;F_{y}\;F_{z}\right]}^{T}$ and contact torques $m^{l}(l)={\left[T_{1}\;T_{2}\;T_{3}\right]}^{T}$ are the initial boundary values of the above differential equations.

The rotation matrix can be specified using Euler angle representation by three successive rotations; first, around the $e_{2}$ axis by an angle of ψ, then the current ${\hat{\text{e}}}_{1}$ axis by θ, and finally the $d_{3}$ axis by φ. Therefore,

(7) $$\left[\begin{array}{c} \dot{\theta }\\ \dot{\psi }\\ \dot{\varphi } \end{array}\right]=\left[\begin{array}{ccc} \cos\varphi & -\sin\varphi & 0\\ \sin\varphi /\cos\theta & \cos\varphi /\cos\theta & 0\\ \sin\varphi \tan\theta & \cos\varphi \tan\theta & 1 \end{array}\right]\left[\begin{array}{c} k_{1}\\ k_{2}\\ \tau \end{array}\right]$$

and

(8) $$\dot{\text{r}}=\left[\begin{array}{c} -\cos\varphi \sin\psi +\sin\varphi \sin\theta \cos\psi \\ \sin\varphi \sin\psi +\sin\theta \cos\varphi \cos\psi \\ \cos\theta \cos\psi \end{array}\right]$$

Numerical integration of Equations (7) and (8) specifies orientation and spatial position of all points and particularly the end point, required in the following section, on the centerline of the catheter. Equations (5) and (6) as an Initial Value Problem (IVP) provide $\left(k_{1},k_{2},\tau \right)$ to be substituted in Equation (7). 

## 3. Kalman Filter-Based Force Observer Design

In many interventions, there are noise contaminations. Moreover, system dynamics and measurement random process properties may vary. Real-time estimation of force imposes another constraint on the efficiency of the solution. Therefore, analytical solutions might not necessarily provide effective solutions. Significantly, stiffness of the catheter might not be available in advance or it could even change during the operation. Kalman filter-based estimators do not require deterministic dynamics or accurate measurements or the random process with stationary properties. In addition to force, stiffness can also be included in the state vector for real-time estimation. Additionally, the noise-filtering advantage of Kalman filters (KFs), as well as their inherent simplicity and effectiveness make KFs well accredited for real applications [29].

In this section, we develop a force estimator based on the adapted model, and compare variants of introduced KF-based estimators with respect to their performance. The proposed scheme allows us not only to provide contact force estimations but also to accommodate the estimates of dominant features causing a catheter’s deformation, e.g., stiffness.

### 3.1. Problem Formulation

#### 3.1.1. Contact Force Estimation (CFE)

For catheter contact force sensing, the desired variables are three-dimensional forces exerted on the catheter tip in local frame. Therefore, we define the state vector of the estimator as follows,

(9) $$X={\left[F_{x}\;F_{y}\;F_{z}\right]}^{T}$$

It has been shown that contact forces can be observed from the catheter shape and deflection [18]. The curvatures, torsion and forces of the segments of the catheter are related through Equations (5) and (6). Consequently, according to Equations (7) and (8), catheter tip pose at time *k*, $T(X_{k})={\left[x_{e,k}\;y_{e,k}\;z_{e,k}\;\theta_{e,k}\;\psi_{e,k}\;\varphi_{e,k}\right]}^{T},$ is a function of applied tip forces. Additionally, regarding corresponding equations, it is concluded that contact forces alter the local curvatures and torsion. Hence, the state space model of the estimator can be formulated as follows,

(10) $$\begin{array}{l} X_{k}=X_{k-1}+\eta_{k}\\ Y_{k}=h(X_{k})+w_{k} \end{array}$$

where the catheter’s current state, $X_{k},$ is assumed to be related to the previous state, $X_{k-1},$ with model uncertainties. The sensor measurements, $Y_{k}$, comprises tip pose and the second-last segment deformations $\omega_{se}={\left[k_{1,se}\;k_{2,se}\;\tau_{se}\right]}^{T}$ as indicated in Figure 2. Here, $\eta_{k}$ and $w_{k}$ are independent zero-mean Gaussian noises that denote uncertainty in states and measurements. Also

(11) $$h(X_{k})={\left[T(X_{k})\;\omega_{se}(X_{k})\right]}^{T}$$

By exploiting the developed model, the Jacobian matrix $J={\left[J_{TF}\;{\text{J}}_{\omega F}\right]}^{T}$ is obtained through calculation of pose (**T**) and deformation ($\omega_{se}$) changes with respect to contact force variations. These matrices can be computed both numerically through approximation with finite differences or analytically with higher accuracy at the expense of more complexity [30].

(12) $$J_{TF}=\left[\begin{array}{cccccc} \frac{\partial x_{e}}{\partial F_{x}} & \frac{\partial y_{e}}{\partial F_{x}} & \frac{\partial z_{e}}{\partial F_{x}} & \frac{\partial \theta_{e}}{\partial F_{x}} & \frac{\partial \psi_{e}}{\partial F_{x}} & \frac{\partial \varphi_{e}}{\partial F_{x}}\\ \frac{\partial x_{e}}{\partial F_{y}} & \frac{\partial y_{e}}{\partial F_{y}} & \frac{\partial z_{e}}{\partial F_{y}} & \frac{\partial \theta_{e}}{\partial F_{y}} & \frac{\partial \psi_{e}}{\partial F_{y}} & \frac{\partial \varphi_{e}}{\partial F_{y}}\\ \frac{\partial x_{e}}{\partial F_{z}} & \frac{\partial y_{e}}{\partial F_{z}} & \frac{\partial z_{e}}{\partial F_{z}} & \frac{\partial \theta_{e}}{\partial F_{z}} & \frac{\partial \psi_{e}}{\partial F_{z}} & \frac{\partial \varphi_{e}}{\partial F_{z}} \end{array}\right]$$

(13) $$J_{\omega F}=\left[\begin{array}{ccc} \frac{\partial k_{1,se}}{\partial F_{x}} & \frac{\partial k_{2,se}}{\partial F_{x}} & \frac{\partial \tau_{se}}{\partial F_{x}}\\ \frac{\partial k_{1,se}}{\partial F_{y}} & \frac{\partial k_{2,se}}{\partial F_{y}} & \frac{\partial \tau_{se}}{\partial F_{y}}\\ \frac{\partial k_{1,se}}{\partial F_{z}} & \frac{\partial k_{2,se}}{\partial F_{z}} & \frac{\partial \tau_{se}}{\partial F_{z}} \end{array}\right]$$

#### 3.1.2. Contact Force and Stiffness Estimation (CFSE)

In order to estimate deflectable distal shaft’s stiffness along with contact force, the contact force and bending stiffness are assumed as the state vector of the estimator as follows:

(14) $$X_{a}={\left[F_{x}\;F_{y}\;F_{z}\;EI_{1}\;EI_{2}\right]}^{T}$$

Shearing stiffness is $GJ_{s}=\frac{E}{2(1+v_{p})}(I_{1}+I_{2}),$ where $v_{p}$ is the Poisson’s ratio. The measurements are considered the same as those presented in Equation (11). Hence, incorporating the state space model specified in Equation (10), the desired states can be estimated. The Jacobian matrix $J_{a}={\left[J_{Ta}\;{\text{J}}_{\omega a}\right]}^{T}$ is determined through calculation of pose (**T**) and deformation ($\omega_{se}$) changes with respect to contact force and stiffness variations. Therefore, $J_{Ta}=\left[\frac{J_{TF}}{J_{TE}}\right]$ and $J_{\omega a}=\left[\frac{J_{\omega F}}{J_{\omega E}}\right]$ are established by the definition of $J_{TE}$ and $J_{\omega E}$ as:

(15) $$J_{TE}=\left[\begin{array}{cccccc} \frac{\partial x_{e}}{\partial EI_{1}} & \frac{\partial y_{e}}{\partial EI_{1}} & \frac{\partial z_{e}}{\partial EI_{1}} & \frac{\partial \theta_{e}}{\partial EI_{1}} & \frac{\partial \psi_{e}}{\partial EI_{1}} & \frac{\partial \varphi_{e}}{\partial EI_{1}}\\ \frac{\partial x_{e}}{\partial EI_{2}} & \frac{\partial y_{e}}{\partial EI_{2}} & \frac{\partial z_{e}}{\partial EI_{2}} & \frac{\partial \theta_{e}}{\partial EI_{2}} & \frac{\partial \psi_{e}}{\partial EI_{2}} & \frac{\partial \varphi_{e}}{\partial EI_{2}} \end{array}\right]$$

(16) $$J_{\omega E}=\left[\begin{array}{ccc} \frac{\partial k_{1,se}}{\partial EI_{1}} & \frac{\partial k_{2,se}}{\partial EI_{1}} & \frac{\partial \tau_{se}}{\partial EI_{1}}\\ \frac{\partial k_{1,se}}{\partial EI_{2}} & \frac{\partial k_{2,se}}{\partial EI_{2}} & \frac{\partial \tau_{se}}{\partial EI_{2}} \end{array}\right]$$

### 3.2. Extended Kalman Filtering

In practice, to deal with system nonlinearities (of state transition and observation functions), extended KF (EKF) is used in many engineering applications [29]. The filter is based on linearization of the nonlinear model with the first order Taylor expansion. 

The EKF-based estimator for both CFE and CFSE is formulated as follows,

(17) $$\begin{array}{l} {\hat{\text{X}}}_{k,k-1}={\hat{\text{X}}}_{k-1}\\ P_{k,k-1}=P_{k-1}+Q_{k}\\ S_{k}=J_{k}P_{k,k-1}{J_{k}}^{T}+R_{k}\\ K_{k}=P_{k,k-1}{J_{k}}^{T}{S_{k}}^{-1}\\ {\hat{\text{X}}}_{k}={\hat{\text{X}}}_{k,k-1}+K_{k}(Y_{k}-h({\hat{\text{X}}}_{k,k-1}))\\ P_{k}=(I-K_{k}J_{k})P_{k,k-1}{\left(I-K_{k}J_{k}\right)}^{T}+K_{k}R_{k}K_{k}^{T} \end{array}$$

Here ${\hat{\text{X}}}_{k}$, $P_{k}$ and $K_{k}$ denote the estimated state for both CFE and CFSE, error covariance matrix and Kalman gain at each time step, respectively. The initial values of the system state vector and error covariance matrix (measured through experiments or initial guess), are represented by ${\hat{\text{X}}}_{0}$ and $P_{0}$, respectively. Significant errors in the initial state estimate, process model, linearization, and covariance matrices may lead to divergence or instability of the estimates in EKFs. 

### 3.3. Iterative and Adaptive Kalman Filters

In order to cope with linearization errors, iterative Kalman filters have been proposed [31], while adaptive filters have been introduced to deal with noise covariance matrix uncertainties [32]. These approaches are concisely presented which can be hierarchically deployed into the structure of nonlinear KF-based estimators.

#### 3.3.1. Iterative Schemes

The iterative filter uses measurements in a recurrent loop to approximate the nonlinear function closely. Additionally, the scheme mitigates the sensitivity to error covariance matrix [31]. More precisely, the prediction equations of the EKF-based estimators are the same in this scheme. However, the *a posteriori* state estimate ${\hat{\text{X}}}_{k}^{l_{i}}$, $l_{i}\leq N$ with ${\hat{\text{X}}}_{k}^{1}={\hat{\text{X}}}_{k,k-1},$ where $l_{i}$ is the number of iteration, is repeatedly calculated using the state estimate of previous iterations. At the end of iterations ${\hat{\text{X}}}_{k}={\hat{\text{X}}}_{k}^{N}$, and the *a* *posteriori* covariance matrix estimate is updated with ${\hat{\text{X}}}_{k}.$

#### 3.3.2. Adaptive Schemes

To deal with uncertainties of model and measurements, adaptive filters are introduced [32,33,34]. Various types of adaptive filters can be adopted. Nonetheless, since the results of different variants are similar, covariance matching is adopted.

The algorithm is based on computing noise statistics for the estimator. The algorithm starts with an initial guess for

(18) $${\hat{\text{q}}}_{k}={\hat{\text{X}}}_{k}-{\hat{\text{X}}}_{k,k-1}$$

(19) $${\hat{\text{r}}}_{k}=Y_{k}-J_{k}{\hat{\text{X}}}_{k}$$

where ${\hat{\text{q}}}_{k}$ and ${\hat{\text{r}}}_{k}$ are the state and measurement noise samples, respectively. These estimates with the mean of noises, ${\bar{\text{q}}}_{k}=\frac{1}{M}\sum_{l_{i}=k-M+1}^{M}{\hat{\text{q}}}_{l_{i}}$ and ${\bar{\text{r}}}_{k}=\frac{1}{M}\sum_{l_{i}=k-M+1}^{M}{\hat{\text{r}}}_{l_{i}}$, over M last samples are implemented in noise covariance estimation. It should be taken into account that larger M leads to more accuracy at the expense of smaller susceptibility to dynamicity, and smaller M results in instability and inaccuracy [34].

The synergy of iterative and adaptive schemes in EKF improves the accuracy and robustness of the filter to nonlinearities and uncertainties of the system. The iterative approach recalculates the *a posteriori* estimate for a specified number of iterations. Afterwards, the adaptive method tunes the noise covariance matrices of the next time step based on previous measurements.

Therefore, the filtering framework starts with an initial guess for $F_{x},F_{y}$ and $F_{z}$. Then Equations (5) and (6) are integrated to provide $k_{1},k_{2},\tau .$ Curvatures and torsion are substituted in Equations (7) and (8). Obtaining the poses leads to specification of curvatures and torsions, $T(X_{k})$ and $\omega_{se}(X_{k})$. Also, $Y_{k}$ is provided through the pose-measuring systems. The provided data are fed into the adopted estimating algorithm and therefore contact forces are estimated at each time step.

## 4. Simulation Results

In order to simulate the proposed model for tri-axial contact force sensing, an ablation catheter (Biosense Webster, Los Angeles, CA, USA) with the geometric and mechanical specifications as indicated in Table 2, is adopted. Simulations are conducted to illustrate and compare the accuracy and robustness of the developed contact force estimators (CFE) for in vivo catheterization, and then to represent the simultaneous contact force and stiffness estimations (CFSE) in required circumstances. At the beginning of the ablation procedure, the stiffness estimation is required while later during the intervention, for more computational efficiency, CFE is preferred.

### 4.1. Accuracy Analysis of CFE 

**Figure 3:**
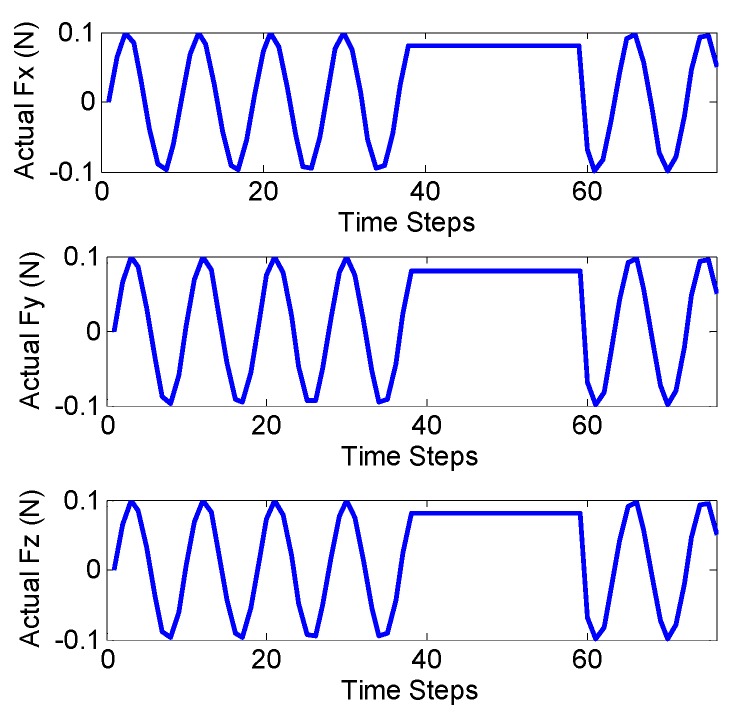
Ground truth of contact force applied to the catheter tip.

**Figure 4:**
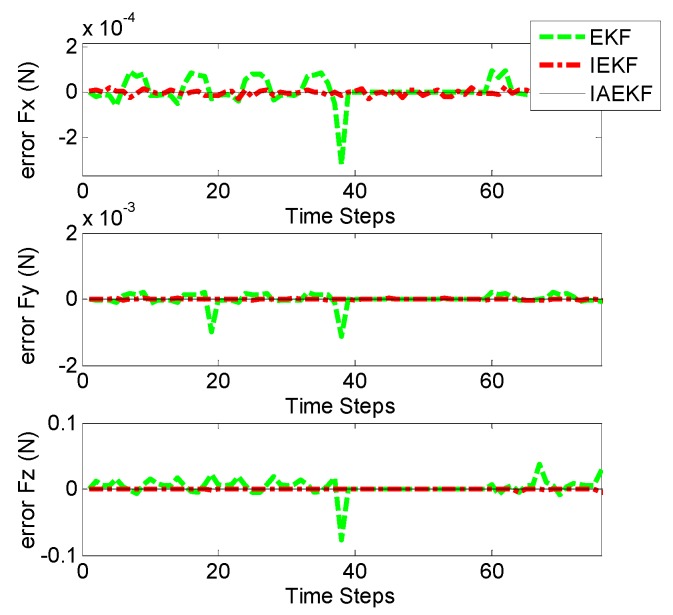
Contact force estimation (CFE) error by EKF-based algorithms with the applied force in all directions including sudden changes.

In the first scenario, the accuracy of the CFE was tested. Tuning nonlinear Kalman filters is not a trivial task. To this end, measurement noise covariance matrix, R = diag(1 mm^2^, 1 mm^2^, 1 mm^2^, 0.0012 rad^2^, 0.0012 rad^2^, 0.0012 rad^2^, 1 mm^−2^, 1 mm^−2^, 1 mm^−2^) was obtained based on the accuracy of measuring device (Aurora^®^ EM Tracking System, NDI, Waterloo, ON, Canada). In the proposed model, Q can be tuned predicting the dynamics of states (which could vary significantly in practice). We chose $\text{Q}=\text{diag}(9\times 10^{-2}\;{\text{N}}^{2},\;9\times 10^{-2}\;{\text{N}}^{2},\;9\times 10^{-2}\;{\text{N}}^{2})$. The human heart ordinarily beats 60–100 times per minute (1–1.7 Hz). Therefore, the designated observers should give reasonable response with sufficient sampling for such a dynamic environment. Figure 3 shows the tri-axial force exerted on the catheter tip including sinusoidal force with the abrupt change to the constant amount to entail regularity and irregularity in cardiac force exertion. Note that the steps represent sampling numbers in the corresponding figures. Figure 4 represents the corresponding errors of force estimation obtained by the EKF-based approaches when the filters are tuned initially. Table 3 gives the detailed statistical values of associated errors of the implemented methods. The results show that EKF does not converge to the ground truth value due to the high nonlinearity of the system as described in Section 3. However, iterative EKF (IEKF) and iterative adaptive EKF (IAEKF) show accurate performance, whereas IAEKF outperforms with the cost of higher computational time. 

**Figure 5:**
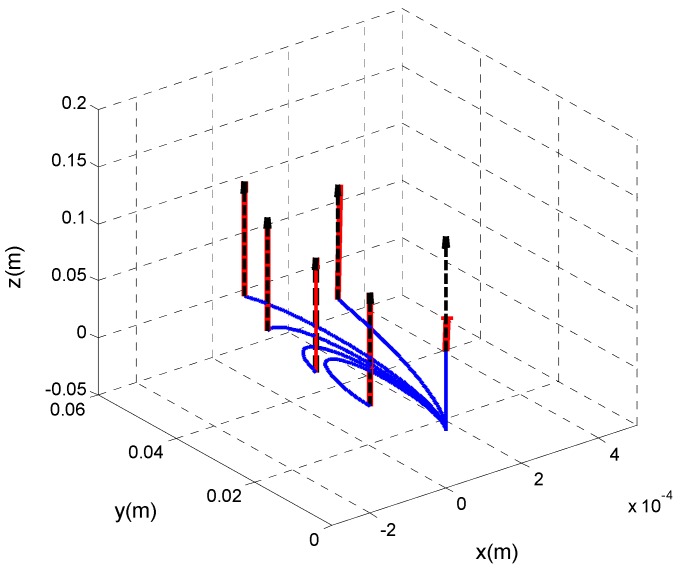
Contact force estimates of the applied forces on the distal tip with the initial curvatures of the distal shaft, $k_{1,0}$, ranging from 0–60 m^−1^. The distal shaft of the catheter is represented in blue, the IEKF-based force estimates are sketched with red arrows, whereas the true values are depicted by black dashed counterparts and are defined in (N).

It should be noted that, when the distal shaft is straight, the axial contact force is ill-sensed. Figure 5 shows the insensible force along $d_{3}$ direction, when the distal shaft is straight. The true applied axial forces along with the estimated ones are represented in Figure 5, when the catheter moves with different initial curvature values, $k_{1,0},$ varying in the range of 0–60 m^−1^. However, since the distal shaft bends passing through the pulmonary vein, this limitation cannot be too restrictive.

### 4.2. Convergence Analysis of CFE

**Figure 6:**
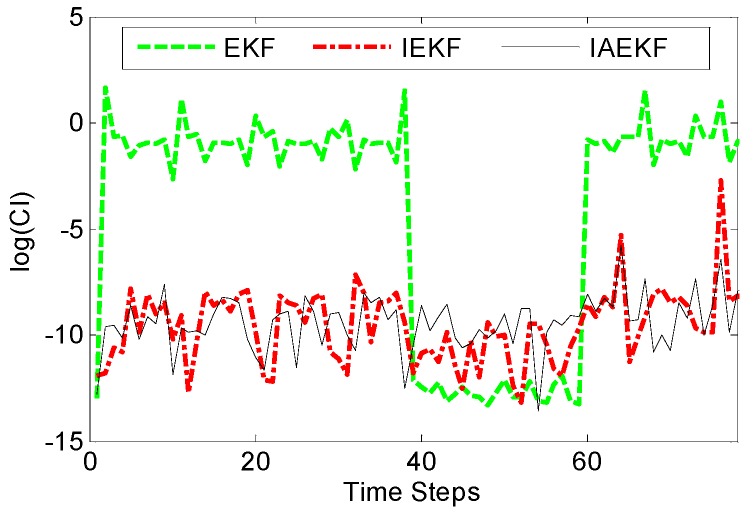
Values of convergence indices (CIs) for CFE by EKF-based approaches.

The performance of a force estimator is usually evaluated according to the errors corresponding to the force estimation. Alternatively, convergence index (CI) can be calculated using the measurement, $\rho_{k}={\hat{\text{r}}}_{k}^{T}S_{k}{\hat{\text{r}}}_{k}$ for EKF-based methods [31]. The smaller the value, the more stable the estimator is. Figure 6 illustrates the convergence of EKF-based approaches for the simulations performed in Section 4.1. It can be seen that IEKF and IAEKF have high convergence, where IAEKF excels in a smaller convergence index.

### 4.3. Sensitivity Analysis of CFE

(1) Measurement noise covariance matrix, $R_{k}$: The convergence of KF-based approaches depends on the measurement noise covariance matrix. Five different measurement noise covariance matrices with diagonal elements $R_{s}\in \{0.02R_{k},\;0.1R_{k},{\text{ R}}_{k},\;10R_{k},\;50R_{k}\}$ with $R_{k}$ defined in Section 4.1, were adopted to study the sensitivity of the method to measurement noises. Table 4 compares the root mean square error (RMSE) for different methods with the applied force along **x** direction, $F_{x}=0.1\sin(2\pi t),$ with 15 samples per second. As it can be seen, IAEKF and IEKF show more robustness to changes of noise covariance matrix.
(2) Initial state estimate, ${\hat{\text{X}}}_{0}$: The cardiac ablation force varies within the range of (0.1–0.4 N) [1]. Therefore, our initial estimates cannot be too far from this range for cardiac-intervention applications. Two sets of initial values, one with 20% (0.2 **F**_max_) and the other one with 50% (0.5 **F**_max_) of the maximum true values, were used as initial estimates. Table 5 compares the corresponding errors of two different initializations for the applied force of $F_{x}=0.1\sin(2\pi t)$.

The sensitivity analyses demonstrate that IEKF and IAEKF have the highest robustness to the filtering uncertainties that might occur during the ablation. 

### 4.4. Contact Force and Stiffness Estimation

**Figure 7:**
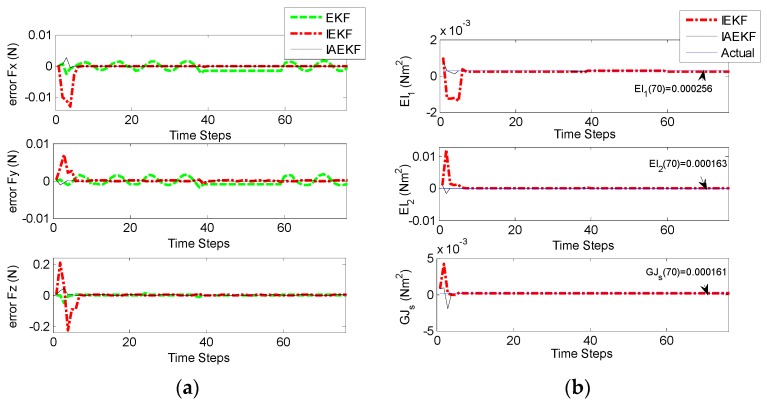
CFSE estimation with the applied tip contact force in all directions including sudden changes by EKF-based algorithms: (**a**) Contact force estimation error; (**b**) Stiffness estimation.

In previous studies, stiffness was assumed to be as an invariant parameter of the system [15,17]. However, this parameter might change as a function of structural specifications of the catheter and different environmental conditions, e.g., environmental temperature [22]. For this purpose, CFSE was tested. The process covariance matrix was assumed to be $\text{Q}\;=\;\text{diag}(9\times 10^{-4}\;{\text{N}}^{2},9\times 10^{-4}\;{\text{N}}^{2},9\times 10^{-4}\;{\text{N}}^{2},10^{-8}\;{\text{N}}^{2}m^{4},10^{-8}\;{\text{N}}^{2}m^{4})$, and R was defined equal to that of CFE in Section 4.1. With the tip contact force represented in Figure 3, the error of force and stiffness estimations are indicated in Figure 7a,b, respectively. The results demonstrate that IEKF and IAEKF provide estimates of contact force with the maximum average error of −0.0016 ± 0.0036 and 0.0008 ± 0.0054, respectively, and stiffness estimates with −1.59 × 10^−4^ and 1.28 × 10^−5^, respectively. The results for EKF-based stiffness estimation due to its divergence have been eliminated. The increased inaccuracy of force estimation is mostly due to high error at the first instants of estimation. Although there is an increased error of contact force estimation compared to that of CFE, the proper choice of measurement vector yielded to convergent estimations for both IEKF and IAEKF. However, depending on the parameters to be estimated, the number of measurement points could be increased.

In the end, the execution times of the proposed approaches are compared. Table 6 illustrates the mean value of computational time of the methods for the simulations. The simulations are performed in MATLAB 2013a^®^ with Core i3 2.2 GHz, 4GB RAM laptop. The results show that IEKF, IAEKF take longer execution time, mostly due to the iterative nature of the schemes. Numerical Jacobian calculation leads to higher computational time that can be compensated by exploiting faster computers, and running the codes in C/C++.

## 5. Experimental Validation

Experiments were designed to compare and verify the performance of the proposed methods. To obtain the stiffness of the catheter, tip forces were applied to the distal shaft and the shape of it was obtained through the measurements of equally spaced point positions 1 cm apart on the distal shaft using CMM machine (Mitutoyo AE112, resolution: 0.001 mm). In our analysis, we considered constant bending stiffness for distal shaft in both directions and $GJ_{s}=0.3846\;E(I_{1}+I_{2})$ with $v_{p}=0.3$. The identification procedure is accomplished by conducting $n_{e}=10$ experiments and solving nonlinear optimization problems for two unknown variables $\left\{EI_{1},EI_{2}\right\}$ by minimizing the distance between measured and model predicted data points as follows:

(20) $$\min\;(J_{\cos t})=\min\;\left(\sum_{j=1}^{n_{e}}\sum_{i=1}^{n_{m}}\left\|r_{\text{model}}(s_{i})-r_{\text{data}}(s_{i})\right\|\right)$$

The optimization was performed using Nelder-Mead simplex method within MATLAB. The results of identification for the ThermoCool SF ablation catheter are presented in Table 2. 

### 5.1. Experiment I

**Figure 8:**
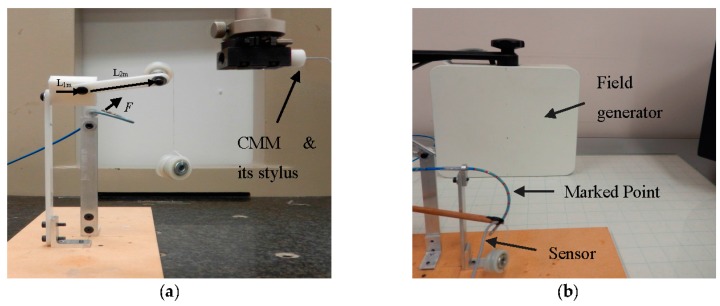
Experimental setup: (**a**) Loading mechanism with two moving links (*L_1m_* and *L_2m_*), pulley and weights; (**b**) Measurement system.

In the first experiment, calibration weights were applied to the catheter tip. Figure 8a represents the experimental setup provided for tri-axial load exertion simulating the forces applied in ablation procedure. The setup consisted of a thread, connected to the tip of the distal shaft, passing through the frictionless pulley with the calibrating weights hung on the other end of the thread. In order to apply spatial forces with the desired direction, the pulley was mounted on the framework with two moving links, as depicted in Figure 8a.

The system in Figure 8b was used to examine the applicability of force estimation approaches. Aurora electromagnetic (EM) tracking system (NDI, Waterloo, ON) consisting of EM field generator and EM tracking sensors provided the position and orientation (pose) of its sensors with the accuracy of 0.48 mm and 0.3°. The curvatures and torsion were obtained through calculating the changes of orientation over arc length in two adjacent end segments of the distal shaft with 4.7 mm distance from one another. Sensor coils were incorporated for measuring the pose of the distal tip and the marked points on the distal shaft as well. Three-dimensional forces were estimated using the measurements and the proposed KF-based approaches.

The catheter distal shaft was bent into two different initial curvatures. The curvature was calculated through marked equispaced point position measurements with 1 cm distance and fitting the optimal constant curvature arc by means of Nelder-Mead simplex method using MATLAB’s *fminsearch* function [35]. In the first scenario of experiment I, planar forces were applied in different directions with the weight 13.03 grams (resembling moderate ablation force of 0.13 N) hung to the pulley and rotating the second link. In the second scenario of experiment I, the spatial forces were applied with the same weight as in previous experiment, but rotating the two links resembling the sinusoidal functions in 3D. The applied forces along the local axes of the distal shaft tip were obtained via pose measurements and obtaining the projected weight forces along the local axes. 

**Figure 9:**
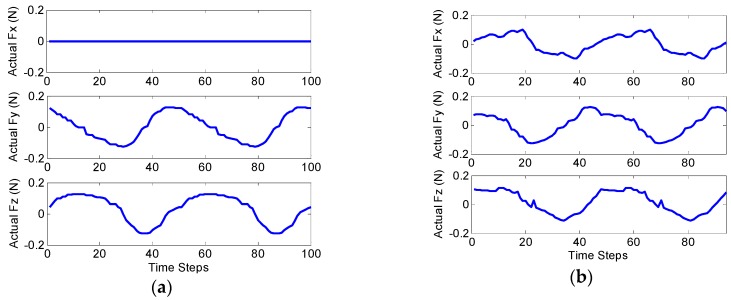
True values of external forces applied to the catheter tip: (**a**) $k_{1,0}=39.99\;{\text{m}}^{-1},$ and quasi-sinusoidal planar force exertion; (**b**) $k_{1,0}=24.57\;{\text{m}}^{-1},$ and quasi-sinusoidal spatial force application.

**Figure 10:**
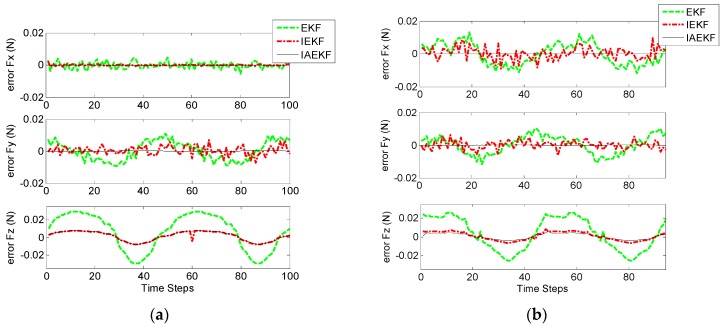
Estimation errors of contact forces applied to the catheter tip: (**a**) $k_{1,0}=39.99\;{\text{m}}^{-1},$ and quasi-sinusoidal planar tip force; (**b**) $k_{1,0}=24.57\;{\text{m}}^{-1},$ and quasi-sinusoidal spatial force.

Figure 9 shows the true values of planar and spatial forces applied to the end of the distal shaft with initial curvatures of $k_{1,0}=39.99\;{\text{m}}^{-1}$ and $k_{1,0}=24.57\;{\text{m}}^{-1},$ respectively. Each experiment was repeated three times for verifying the repeatability of the introduced methods. Figure 10a,b represent the corresponding errors of estimation for aforementioned contact forces performed by EKF, IEKF, and IAEKF. For better comparison of the results, the average error values and corresponding standard deviation of estimations are summarized in Table 7. The entries of the measurement covariance matrix were taken to be 1000 times that of the determined $R_{k}$ to account for the measurement uncertainty due to high sensitivity of the EM tracking system to trivial distortions. The results show that IAEKF has the highest accuracy with the maximum average error value along axial direction with the precision of 0.001 ± 0.0041 N. However, the force estimations provided by IEKF and IAEKF are comparable with the existing force sensors [11] and estimators [18]. 

### 5.2. Experiment II

**Figure 11:**
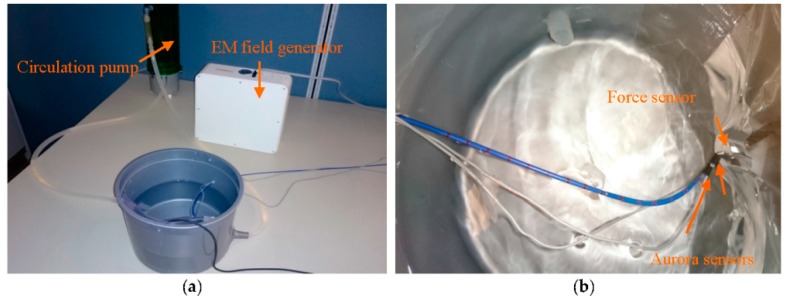
Experimental apparatus mimicking the intra-cardiac chamber with circulating flow for CFE: (**a**) The setup consisting of EM tracking system, circulation pump, ablation catheter and the force sensor; (**b**) Close-up view of the catheter and the EM sensors attached to it interacting with the force sensor.

In the second set of experiments, CFSE and CFE were evaluated in a simulated environment of intra-cardiac catheterization. For CFE, the setup in Figure 11a was prepared. The water circulation pump (EHIEM, Deizisau, Germany) circulated the saline water in the bucket mimicking the blood flow in intra-cardiac chambers. The force sensor (Nano 17, ATI, Apex, NC, USA) was attached to the bucket wall to interact with the catheter tip while measuring the tri-axial contact forces. The catheter was bent with initial curvature of $k_{1,0}=19.41\;{\text{m}}^{-1}.$ Two Aurora sensors were attached to the catheter distal shaft to provide tip pose, curvatures and torsion of the second-last segment required for force sensing as illustrated in Figure 11b. The sensors were placed 4.7 mm apart and calibrated to measure the desired poses of the two end segments.

**Figure 12:**
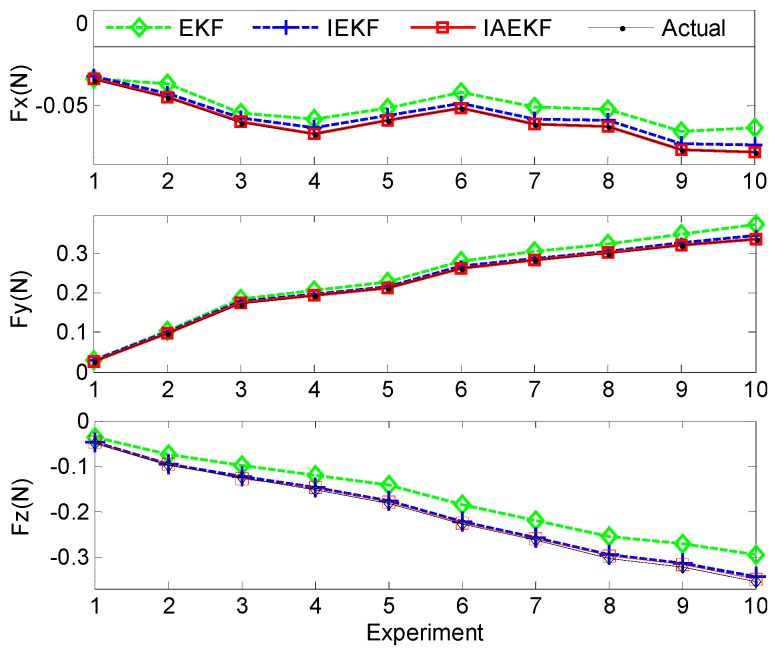
Estimated forces compared with true values for different implemented contact forces in Experiment II with the initial curvature of $k_{1,0}=19.41\;{\text{m}}^{-1}.$

In this experiment, the bucket with the force sensor was moved forward manually toward the distal shaft base, and the force estimation was performed for the sampled data points. Figure 12 illustrates the applied and estimated spatial contact forces expressed in the local distal shaft frame coordinates. In the second set of experiments, the measurement covariance matrix was assumed to be equal to that of Experiment I, except the last three diagonal elements when it was assumed to be twice those of the covariance matrix due to uncertainties imposed by circulating water over the curvature and torsion measurements of the second-last segment. In addition, the estimations for the sampled data points were repeated five times. The results show the consistency with the previous experiments and simulations, where the IAEKF estimator has the maximum average error of 0.0028 ± 0.0019 N along the axial direction. The average errors of estimations are summarized in Table 7.

**Figure 13:**
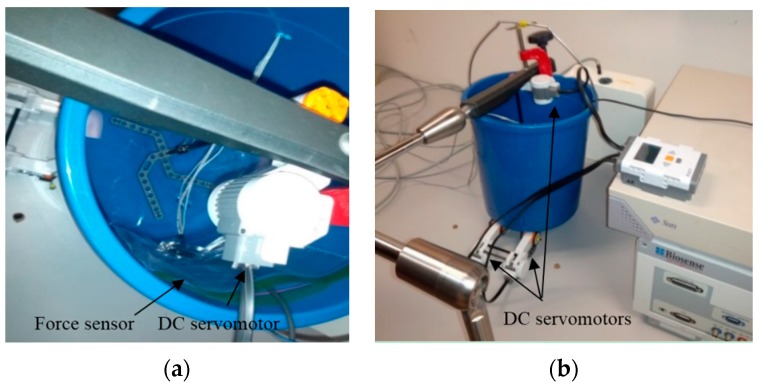
Experimental apparatus mimicking an intra-cardiac chamber for CFSE: (**a**) Close-up view of the setup consisting of EM tracking system, a motor for fluid circulation, ablation catheter and the force sensor; (**b**) Two dc servomotors translating the aforementioned assembly for making the dynamic interacting load.

In the second scenario, the setup in Figure 13 was prepared for CFSE. To simulate the beating heart as the dynamic interacting contact load of the distal shaft, two DC servomotors (The Lego Group, Billund, Denmark), a bucket accommodated on top of the motors, and the force sensor attached to the bucket wall, as represented in Figure 13a,b, were incorporated. The distal shaft tip was in contact with the force sensor and the motors were connected together and were moving the bucket. The bucket was translated with a frequency and sampling time of approximately 0.21 Hz and 0.3 s, respectively, to comply with the computational requirements of CFSE framework. However, the computational costs easily can be reduced as mentioned in Section 4. In cardiac catheterization, the blood flow has the most significant effect in making disturbances in both sensor readings and process model. In this scenario, water was circulated with the other DC servomotor as illustrated in Figure 13a. Force and EM sensors were calibrated and attached to the bucket wall and distal shaft, respectively, as in the first scenario of Experiment II.

**Figure 14:**
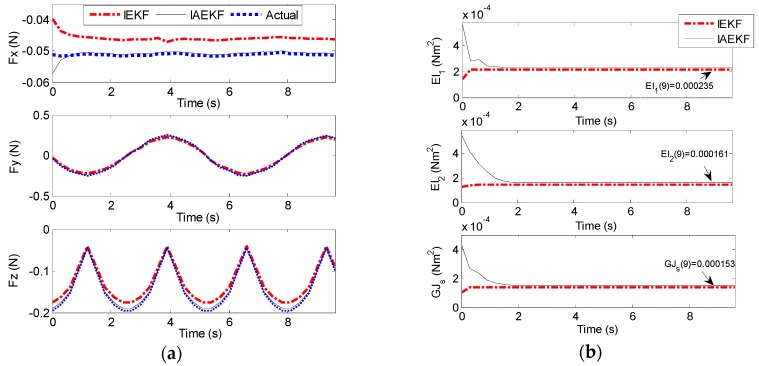
CFSE estimation with the applied tip contact forces using the setup in Experiment II (b): (**a**) Contact force estimation; (**b**) Stiffness estimation.

Figure 14a,b represent the results of contact force and stiffness estimation of distal shaft interacting with the moving force sensor. There is no direct way for evaluation of the accuracy of stiffness estimation in different operational conditions. However, it can be assessed indirectly from the accuracy of force estimation. The maximum average error of force estimation for IEKF and IAEKF are 0.0138 ± 0.0050 N and 0.0035 ± 0.0012 N, respectively, where the IAEKF estimator due to its adaptability has the highest accuracy and robustness to the uncertainties. Despite shortcomings of the experiment (i.e., ignoring cardiac tissue elasticity, catheter’s pathway from pulmonary vein to the atrial and compliance of blood vessels connected to the heart), it provides good basis for proof of concept. It should also be noted that this is the first study considering simultaneous force and stiffness estimation.

The primary sources of errors in the experiments are the pose measuring system and consequently deformation calculation inaccuracy, and discrepancy of evaluated initial curvatures from the true values. The former one can be tackled with the exploitation of more accurate measuring system and imaging modalities, while it can relax the inaccuracy of distal shaft initial curvature estimation as well. In addition, incorporating redundant pose measurements along the distal shaft can lead to more precise initial curvature attribution. In clinical applications, image-based pose measurements would be feasible. Modeling uncertainty is the other source of error. The model can be elaborated by considering the contact forces and tendon loadings acting along the distal shaft. However, the computational time and accuracy tradeoff should be taken into account. 

## 6. Discussion

In intra-cardiac catheterization procedures’ force measurements are very helpful for the success of both manual and robot-assisted catheterization. A new framework for tri-axial shape-based contact force and stiffness estimation of tendon-driven catheters was introduced in this paper. The introduced estimators show the potential of Kalman filter (KF)- and observer-based methods as the alternative for integrating often costly and inconvenient catheter tip force sensors. In particular, readily available images during intervention can be used to estimate the curvatures and position of required points on the catheter for the proposed method. It has been demonstrated that contact forces can be observed using catheter deformation [18]. 

In this work, nonlinear Kalman filters utilizing tip pose and curvature measurements were formulated for force and stiffness estimation. The shape-based force estimators were developed based on the accurate Krichhoff rod model of the tendon-driven catheter. To cope with model and measurement nonlinearities and uncertainties, iterative and adaptive structures were integrated into EKF formulation to provide reliable force and stiffness estimators. The IAEKF approach increases the accuracy and robustness of the estimation considerably at the expense of more computational time. Implementation of iterative approaches requires tuning of iteration numbers, since over-iterating results in higher computational time. The window size of adaptive filters should be adjusted as well, since its improper size would result in the instability of the system. The simulation and experimental results show the potential application of the proposed method in clinical experiments. For future work, we intend to perform in vivo tests. For achieving more accurate and redundant pose measurements, we plan to use medical imaging modalities along with EM tracking sensor measurements. The filtering framework is expected to be elaborated upon in the presence of point and distributed forces along the distal shaft taking into account changing contact forces as a result of varying heart beat rate in cardiac arrhythmia treatment. Furthermore, we plan to incorporate measurements of tissue deformation in the case of zero curvature of the distal tip. 

## Figures and Tables

**Table 1:** Nomenclature.

$d(s)=\left\{d_{1}(s),d_{2}(s),d_{3}(s)\right\}$	Local Coordinate Frame
$e=\left\{e_{1},e_{2},e_{3}\right\}$	Global coordinate frame
E, $I_{j}$, EI	Young’s modulus, second moment of area about $d_{j}$ (*j* = 1, 2), bending stiffness
f(s)	External body force at point *s*
F=[*F_x_*,*F_y_*,*F_z_*]^T^	Distal tip contact force vector
G, $J_{s}$, $GJ_{s}$	Shear modulus, polar moment of inertia, shearing stiffness
J	Jacobian matrix
$k_{1},k_{1,0}$	Curvature about $d_{1}$, initial curvature about $d_{1}$
$k_{2},k_{2,0}$	Curvature about $d_{2}$, initial curvature about $d_{2}$
l(s)	External body moment at point *s*
$l_{i}$	Number of iterations
*L*	Distal shaft length
m(s)	Internal moment vector at point *s*
*M*	Window size
n(s)	Internal force vector at point *s*
$\hat{\text{q}}$	Process noise sample vector
Q	Process noise covariance matrix
r(s)	Centerline position in global frame
$\hat{\text{r}}$	Measurement noise sample vector
R	Measurement noise covariance matrix
$R_{c}(s)$	Rotation matrix at point *s*
v	Velocity vector
w	Measurement noise
$X,\;{\text{X}}_{a}$	State vector of the estimator for CFE and CFSE
Y	Measurement vector of the filter
η	Process noise vector
θ, φ, ψ	Euler angles representing rotation of points on centerline
$\tau ,\tau_{0}$	Torsion about $d_{3}$, initial torsion about $d_{3}$
ω	Darboux vector
$\omega_{se}$	Second-last segment deformation vector

**Table 2:** Specifications of cardiac ablation catheter.

Distal Shaft Parameters	Value
Length	70 mm
Diameter	7 F (2.33 mm)
Bending stiffness $EI_{1}$ and $EI_{2}$	0.2612 × 10^−3^, 0.16664 × 10^−3^ Nm^2^
Shearing stiffness $GJ_{s}$	0.1645 × 10^−3^ Nm^2^

**Table 3:** Comparison of EKF-based estimators for the applied spatial forces (N).

		$e(F_{x})$	$e(F_{y})$	$e(F_{z})$
EKF	MAX (ABS)	3.237 × 10^−4^	0.0011	0.0774
	MEAN	1.124 × 10^−5^	−7.355 × 10^−6^	0.0039
	STD	5.521 × 10^−5^	1.859 × 10^−4^	0.0122
IEKF	MAX (ABS)	3.147 × 10^−5^	3.992 × 10^−5^	0.0048
	MEAN	1.957 × 10^−6^	1.070 × 10^−6^	2.013 × 10^−4^
	STD	1.209 × 10^−5^	1.361 × 10^−5^	7.917 × 10^−4^
IAEKF	MAX (ABS)	1.436 × 10^−7^	6.957 × 10^−7^	2.488 × 10^−5^
	MEAN	9.747 × 10^−9^	4.560 × 10^−8^	1.480 × 10^−6^
	STD	3.147 × 10^−8^	1.436 × 10^−7^	6.814 × 10^−6^

**Table 4:** RMSE (N) of KF-based force estimation techniques for $F_{x}=0.1\sin(2\pi t)$ with different values of $R_{k}$.

	0.02 $R_{k}$	0.1 $R_{k}$	$R_{k}$	10 $R_{k}$	50 $R_{k}$
EKF	0.0164	0.0160	0.0144	0.0113	0.0079
IEKF	3.237 × 10^−6^	1.304 × 10^−6^	6.849 × 10^−6^	2.216 × 10^−5^	4.257 × 10^−5^
IAEKF	2.878 × 10^−7^	4.471 × 10^−7^	1.296 × 10^−6^	1.539 × 10^−5^	4.641 × 10^−5^

**Table 5:** RMSE comparison of EKF-based force estimates for two different force initialization: 0.2 and 0.5 of maximum ground truth.

		$e(F_{0.2x_{\max}})/e(F_{0.5x_{\max}})$	$e(F_{0.2y_{\max}})/e(F_{0.5y_{\max}})$	$e(F_{0.2z_{\max}})/e(F_{0.5z_{\max}})$
EKF	MEAN	−3.17 × 10^−7^/−1.01 × 10^−5^	−1.11 × 10^−5^/−3.19 × 10^−5^	−0.0102/−0.0273
	STD	1.054 × 10^−7^/2.5 × 10^−5^	1.95 × 10^−5^/3.83 × 10^−5^	0.0091/0.0170
IEKF	MEAN	2.93 × 10^−7^/2.72 × 10^−6^	8.90 × 10^−8^/−1.80 × 10^−6^	4.06 × 10^−5^/2.06 × 10^−6^
	STD	2.19 × 10^−6^/2.04 × 10^−6^	3.60 × 10^−6^/3.63 × 10^−6^	1.70 × 10^−4^/1.74 × 10^−4^
IAEKF	MEAN	−6.63 × 10^−9^/2.70 × 10^−9^	−3.19 × 10^−8^/2.08 × 10^−8^	−2.13 × 10^−5^/−3.12 × 10^−5^
	STD	6.056 × 10^−8^/1.06 × 10^−8^	2.11 × 10^−7^/1.98 × 10^−7^	3.04 × 10^−4^/1.91 × 10^−4^

**Table 6:** Computational time for EKF-based estimates with $l_{i}=10,\;M=15.$

Method		Time(s)
CFE	EKF	0.029
	IEKF	0.11
	IAEKF	0.175
CFSE	EKF	0.049
	IEKF	0.176
	IAEKF	0.28

**Table 7:** Average force estimation errors (N) for EKF, IEKF and IAEKF in experiments.

		Experiment I (a)			Experiment I (b)			Experiment II (a)		
		$e(F_{x})$	$e(F_{y})$	$e(F_{z})$	$e(F_{x})$	$e(F_{y})$	$e(F_{z})$	$e(F_{x})$	$e(F_{y})$	$e(F_{z})$
EKF	MEAN	1.03 × 10^−4^	3.09 × 10^−4^	6.5 × 10^−3^	2.71 × 10^−4^	2.38 × 10^−4^	0.003	9.4 × 10^−3^	0.0181	0.0375
	STD	0.002	5.8 × 10^−3^	0.0202	6.9 × 10^−3^	5.4 × 10^−3^	0.0174	2.4 × 10^−3^	9.8 × 10^−3^	0.0133
IEKF	MEAN	3.86 × 10^−5^	5.88 × 10^−4^	1.6 × 10^−3^	4.86 × 10^−4^	2.82 × 10^−4^	7.65 × 10^−4^	0.003	4.8 × 10^−3^	0.0055
	STD	4.23 × 10^−4^	2.8 × 10^−3^	5.4 × 10^−3^	3.7 × 10^−3^	2.6 × 10^−3^	4.5 × 10^−3^	7.01 × 10^−4^	1.4 × 10^−3^	0.002
IAEKF	MEAN	6.01 × 10^−6^	5.03 × 10^−5^	1.7 × 10^−3^	4.38 × 10^−6^	9.26 × 10^−5^	3.10 × 10^−4^	7.39 × 10^−5^	5.42 × 10^−4^	0.0028
	STD	4.36 × 10^−6^	9.67 × 10^−5^	5.2 × 10^−3^	1.06 × 10^−5^	1.65 × 10^−4^	0.003	5.55 × 10^−5^	2.70 × 10^−4^	0.0019

## Abbreviations

The following abbreviations are used in this manuscript:

RF	Radiofrequency
MRI	Magnetic resonance imaging
CI	Convergence index
CFE	Contact force estimation
CFSE	Contact force and stiffness estimation
KF	Kalman filter
EKF	Extended Kalman filter
IEKF	Iterative extended Kalman filter
IAEKF	Iterative adaptive extended Kalman filter
EM	Electromagnetic
RMSE	Root mean square error
